# Dual Stressors and Female Pre-school Teachers' Job Satisfaction During the COVID-19: The Mediation of Work-Family Conflict

**DOI:** 10.3389/fpsyg.2021.691498

**Published:** 2021-06-08

**Authors:** Xiumin Hong, Qianqian Liu, Mingzhu Zhang

**Affiliations:** Faculty of Education, Institute of Early Childhood Education, Beijing Normal University, Beijing, China

**Keywords:** work overload, parenting stress, work-family conflict, job satisfaction, the COVID-19 epidemic

## Abstract

Online education has become a vital weapon to fight against the COVID-19 epidemic in the world. In the home-based online education environment, female pre-school teachers are expected to balance the dual roles of teacher and mother at the same time, which may trigger the work-family conflict. Although previous studies analyzed individual stressors, work-family conflict and its outcomes, there is little research on pre-school teachers' work and parenting experience during major public health emergencies. The current study examined the associations among work overload, parenting stress, work-family conflict, and job satisfaction during the COVID-19. Seven hundred eighteen female pre-school teachers with children who worked online at home participated in the study. Female pre-school teachers reported that the COVID-19 has increased work overload and parenting stress. Moreover, work overload was negatively associated with job satisfaction via its positive association with work-to-family conflict. Parenting stress was negatively associated with job satisfaction via both family-to-work conflict and work-to-family conflict. The study contributes to a better understanding of the association among female pre-school teachers' work overload, parenting stress, work-family conflict, and job satisfaction. Our findings highlighted potential avenues for interventions aimed at balancing female pre-school teachers' work and family and improving their job satisfaction during the COVID-19.

## Introduction

The COVID-19 pandemic is first and foremost a health crisis and then quickly expanded to the economic, social life, and education sector of the global world (Thu et al., 2020). In the education sector, China closed down pre-schools, schools, and universities, and the Ministry of Education carried out the initiative of “suspension of classes without suspension” during the COVID-19 (Ministry of Education, 2020). This initiative means that children and teachers are forced to stay at home, changing the way they learn and teach. When the educational place is transferred to the home, work and family processes are in the same space, which blurs the boundary between the work and family field, and ultimately leads to work-family conflict more easily. It has long been a challenge for women in the workplace, such as female teachers, especially those with children. A large number of studies have shown that female teachers face the challenge of work-family conflict (Noor and Zainuddin, 2011; Erdamar and Demirel, 2014).

During the COVID-19 crisis, the dual stressors of work and family make the work-family conflict more obvious. Female pre-school teachers with children are a special group, who shoulder the dual role of teacher and mother. As a pre-school teacher, she has to educate other people's young children (job demands). As a mother, she needs to accompany herself with their children at home (family demands). If the two fields are not handled properly, there will be an imbalance, and it may also bring a series of negative consequences. Research has examined the work-family conflict is a risk factor for job satisfaction (Buonocore and Russo, 2013). Although existing research has focused on role stressors, work-family conflict, and job satisfaction (Bakker et al., 2011; Beham et al., 2011), there is little research on pre-school teachers' work-family conflict and its outcomes during the major public health emergency. Teacher job satisfaction is an important topic in China. The research suggests that Chinese pre-school teachers' job satisfaction is low and it is necessary to take measures to improve it (Jiang et al., 2019). In the context of the COVID-19 epidemic sweeping the world, Chinese teachers' job satisfaction may be influenced by dual role overload and work-family conflict, which remains to be studied.

The present study seeks to address this gap by investigating an array of perceived dual stressors (i.e., work overload and parenting stress) reported by female pre-school teachers during COVID-19. Also, we sought to understand the relationships among pre-school teachers' work overload, parenting stress, work-family conflict, and job satisfaction. Results have the potential to reveal indicators for pre-school teachers that may induce dissatisfaction and burnout in the teaching profession. Besides, this information may be particularly valuable in improving teacher well-being in China and around the world during special periods. For example, pre-school education managers could use this information to improve training courses more purposefully. These and other practices could equip pre-school teachers with the skills to balance work and family, potentially setting them on more positive career trajectories during COVID-19.

## Literature Review

### Teaching Force of Pre-school in China During the COVID-19

Pre-school Teachers' Professional Standards (Ministry of Education, 2012), a national professional standard, has made definite requirements on Chinese pre-school teachers' belief, knowledge, and ability. Meanwhile, the state also requires that candidates must finish vocational university or above and obtain pre-school teacher certification. Unfortunately, in the reality, pre-school teacher qualifications have not always been able to keep up. 17.3% of pre-school teachers in China have not obtained a vocational university degree or above (Ministry of Education, 2019). A recent study found that 12.2% of pre-school teachers did not major in pre-school education, and about half of them (50.4%) had few opportunities participated in high-quality training organized by the central government (Hong and Zhang, 2020).

The sudden emergence of online education may bring professional challenges for pre-school teachers during the COVID-19. The novel coronavirus has spread very rapidly all over the world and has aroused people's concern (Bao et al., 2020). Facing the unprecedented, unexpected, and threatening natural disaster, China immediately launched the crisis response mechanism (Xue et al., 2020). In the field of pre-school education, the Ministry of Education carried out the national initiative, i.e. “suspension of classes without suspension” (Ministry of Education, 2020). The classroom was moved from pre-school to home, which breaks the traditional teaching model with classroom and class as the boundary and has higher requirements on teachers' ability of home-based online education. To support the young children's development during the epidemic, pre-school teachers should not only quickly adapt to the use of information technology to carry out daily educational activities, but also undertake inexperienced teaching contents, such as statistics of children's health information and epidemic education. These tasks require pre-school teachers to handle remotely anytime, which has added a lot of burdens and work stress for pre-school teachers, and prolonged their working hours.

When the workplace was transferred to the family, in addition to work role overload, pre-school teachers may also face family role stress, especially for pre-school teachers with children. Influenced by the traditional Chinese labor division of “men do farm work and women do housework” (Xu, 2016), professional women still need to spend more time and energy raising children. During the COVID-19, mothers have more frequent and longer contact with their children, which would significantly increase pre-school teachers' parenting stress in-home quarantine. The relentless requirement of managing other people's young children and their children at the same place may exacerbate the conflict between work and family because this demanding task is central to both roles. In sum, dual role overload of work and parenting may lead to high levels role of conflict for pre-school teachers. As such, investigating pre-school teachers' work and parenting experiences could provide further insights into pre-school teachers' working and living conditions in the special working context.

Pre-school education is the most basic education, and it offers a solid foundation for formal education. Pre-school teachers are expected to display more pleasurable emotions and higher quality professional competence during work to cultivate children's secure attachment, social-emotional competence, and cognitive development (Denham et al., 2012; Fuhs et al., 2013; Wang et al., 2020). However, it is not so easy for pre-school teachers to maintain the generally positive work condition if they are experiencing enormous work overload, parenting stress and work-family conflict, and subsequent low job satisfaction. Low levels of job satisfaction can have negative consequences for psychological and physical health (Faragher, 2005). Specifically, it can have negative consequences for pre-school teachers' instructional quality, ultimately hampering children's successful development (Veldman et al., 2013; Klusmann et al., 2016). Our study may provide enlightenment for improving pre-school teachers' occupational well-being and even young children's outcomes during the epidemic.

### Model and Hypothesis

#### Work-Family Conflict

Work-family conflict is a form of inter-role conflict where the role pressures from the work and family domains are mutually incompatible in some respects (Greenhaus and Beutell, 1985). Boundary theory (Ashforth et al., 2000) and conservation of resources theory (Hobfoll, 1989) propose that when individuals perceive the role overload in a certain field, they would reallocate their resources among different roles to realize the interdomain transition. Therefore, when an individual realizes that he or she does not have enough time and energy to complete a task in a certain field, he or she will tend to use the original allocation to the resources of another domain (such as time or energy), causing conflicts between the two domains (Matthews et al., 2013). For example, allocating too many resources to work could hinder the performance of pre-school teachers' parenting responsibilities. Conversely, the overabundance of parenting resources disturbs the achievements of their work-related tasks. The former denotes that the requirements of the work domain hinder the needs of the family, i.e., work-to-family conflict, and the latter means that the demands of the family field impede the demands of work, i.e., family-to-work conflict (Netemeyer et al., 1996; Frone et al., 1997; Frone, 2003).

Frone et al. (1992) proposed and tested the work-family conflict model that has dominated the literature since its publication (Bellavia and Frone, 2005). The model hypothesized a positive reciprocal relationship between work-to-family conflict and family-to-work conflict. The model is based on the assumption that if individuals' work-related problems begin to interfere with the accomplishment of individuals' family-related responsibilities, these unfulfilled family responsibilities may begin to interfere with one's daily functioning at work. Conversely, the unfulfilled work obligations may begin to interfere with functioning at home, when individuals' family-related stress starts to interfere with the accomplishment of one's work-related obligations. This proposed hypothesis is consistent with Schaubroeck (1990) suggestion that the conflicted relationship between work and family may be examined appropriately within the context of a bidirectional relationship. A study showed that as work-to-family conflict increased, family-to-work conflict increased, and vice versa, and this relationship held up over time (Huang et al., 2004). Subsequently, we proposed the following hypothesis:

H1. Pre-school teachers' work-to-family conflict and family-to-work are positively associated with each other.

#### Antecedents of Work-Family Conflict: Work Overload and Parenting Stress

Work-to-family conflict and family-to-work are different and caused by different stressors (Mesmer-Magnus and Viswesvaran, 2005). More specifically, work-to-family conflict is mainly associated with work-related stress, while family-to-work conflict is mainly associated with family-related stress (Bakker et al., 2011). In the work domain, work-related factors such as job stress and work hours create work-to-family conflict (Ismail and Gali, 2016; Allen et al., 2020). Previous studies strongly supported the positive relationships between work overload and work-to-family conflict (Bakker et al., 2011; Beham et al., 2011; Armstrong et al., 2015; Lambert et al., 2017; Olaniyan, 2020). A study of a sample of 337 Chinese pre-school teachers also found that work overload can increase work-to-family conflict (Gu and Wang, 2019).

For family-to-work conflict, the family-related stress is the main antecedent variable. However, in the field of the teaching profession, most of the studies investigating teachers' work-family conflict only focus on the influence of work-related stressors and pay little attention to the stressors in family life (i.e., parenting stress). Parenting stress is an important source of family stress, especially for working women with children. A limited number of studies have demonstrated the relationship between child-rearing-related stress and family-to-work conflict. For example, a study based on New Zealand teachers with dependent children found that children's behavior problems increased family-to-work conflict (Palmer et al., 2012).

During the COVID-19, pre-school teachers may experience the dual stress of work and parenting and they need to balance the roles of staff and mothers at home in the long term (Limbers et al., 2020). Although there is a bilateral relationship between work-family conflict, the existing studies look at it as a whole (Gopalan and Pattusamy, 2020). As such, this present study focused on both the association between work overload and work-to-family conflict and the association between parenting stress and family-to-work conflict. Compared with previous studies that only focused on pre-school teachers' job-related stress, our study on the dual stressors provides a more comprehensive explanation of the stressors of pre-school teachers' work-family conflict. Based on the previous literature, the study proposed the following hypotheses:

H2. Pre-school teachers' work overload is positively associated with work-to-family conflict.

H3. Pre-school teachers' parenting stress is positively associated with family-to-work conflict.

#### Consequences of Work-Family Conflict: Job Satisfaction

Work-family conflict is associated with many negative consequences, including work-related, family-related, and other domain results (Bellavia and Frone, 2005; Matthews et al., 2014). In the work field, job satisfaction is a very important outcome variable in the work-family conflict model developed by Frone (Amstad et al., 2011). Job satisfaction is the pleasant or positive emotional state that employees have for their jobs (Judge et al., 2017). This sense of satisfaction is helpful to reduce pre-school teachers' turnover intention and improve their work performance (Liu and Ramsey, 2008), which is ultimately beneficial to the development of young children.

Work-to-family conflict and family-to-work conflict are associated with job satisfaction to some extent. Based on the literature review, there are two views about these associations: cross-domain relations vs. matching-domain relations.

Frone et al. (1992) assumed a cross-domain relationship in their work-family conflict model, implying that family-to-work conflict mainly affects the work domain. The rationale behind this assumption is that the conflict, although originating in one domain, is causing problems in the other domain. As a consequence, well-being related to this other life domain suffers. For example, individuals experiencing family-to-work conflict cannot spend as much time on their work as they would like because of their family responsibilities. As a consequence, job performance suffers, inducing work-related outcomes (e.g., job satisfaction) to decrease.

Different from Frone's viewpoint, Amstad et al. (2011) proposed a “matching-hypothesis” based on a meta-analysis of existing literature, which assumes that the primary effect of work-family conflict lies in the domain where the conflict originates. According to this hypothesis, work-to-family conflict should have stronger effects on work-related outcomes. The rationale for this assumption refers to appraisal, most notably attributional, processes. Based on attribution theory, the cause of work-to-family conflict is seen in one's work (Martinko and Mackey, 2019). People are likely to dwell on the causes of work-to-family conflict, their characteristics, their consequences, ways of dealing with them, and so forth. To the extent that this occurs, people's thoughts are likely to be centered around the work situation and would blame the work, which in turn trigger negative emotions toward the job, thus leading to a decrease in job satisfaction. In sum, negative affective reactions are likely to center around the domain that is seen as causing the problem, implying that strain reactions should be dominant about this domain.

Interestingly, the two views mentioned above are not contradictory. That is, work-to-family conflict may be associated with job satisfaction, and family-to-work conflict may also harm job satisfaction. However, limited studies combine two theoretical perspectives. Thus, based on the prior research, the present study projected the following hypotheses:

H4. Pre-school teachers' work-to-family conflict is negatively associated with job satisfaction.

H5. Pre-school teachers' family-to-work conflict is negatively associated with job satisfaction.

#### Associations Among Work Overload, Parenting Stress, Work-Family Conflict, and Job Satisfaction

Researchers have considered the direct and indirect associations among work overload, parenting stress, work-family conflict, and job satisfaction. About direct relationships, work-related and nonwork-related stressors (such as parenting stress) are associated with job satisfaction (Scanlan and Still, 2019). As we just mentioned, empirical studies found that work overload was strongly related to job satisfaction. Low perceptions of work overload may have contributed positively to one's assessments of their job satisfaction. Family-related stress was the risk factor of professional experience (Cao et al., 2020). Parenting stress may arise from a parent's appraisal of contextual demands associated with the parenting role, such as insufficient personal resources to meet the demands of caring for their children. To the extent that parenting stress is linked to one's depression and distress (Rollè et al., 2017), it is also likely to be linked to lower job satisfaction. If strain from the parenting role takes its toll on the parents, parents may not be able to enjoy the work role as much as they would if they had the energy and time.

What's more, previous research has documented work-family conflict as a mediator between stressors and outcomes, such as job satisfaction (Love et al., 2010). Considering that there are two relations between work-family conflict and job satisfaction (i.e., cross-domain relations and matching-domain relations), existing studies mainly include two aspects. On the one hand, previous research has confirmed the matching-domain effect of work-family conflict. For example, Rubel et al. (2017) reported that work-to-family conflict plays an indirect role between work-related overload and job outcomes (i.e., seek new employment). On the other hand, following the opinions of the cross-domain hypothesis, family-to-work conflict could mediate parenting stress and job satisfaction. This assumption has received empirical support in several studies. A meta-analysis by Ford et al. (2007) showed that family-to-work conflict plays an indirect role between family stress and job satisfaction, despite the direct effect of family stress on job satisfaction being not found. It is also notable that the above studies focus on the field of management, and empirical research is needed to support whether they are applicable to teachers, especially pre-school teachers in the context of the epidemic. Also, previous studies have focused on considering cross-domain and matching domain relationships separately, while limited studies have integrated the two viewpoints. Therefore, it is theorized that:

H6. Work overload is negatively associated with and job satisfaction via a positive association with work-to-family conflict.

H7. Parenting stress is negatively associated with and job satisfaction via a positive association with family-to-work conflict.

**Figure 1:**
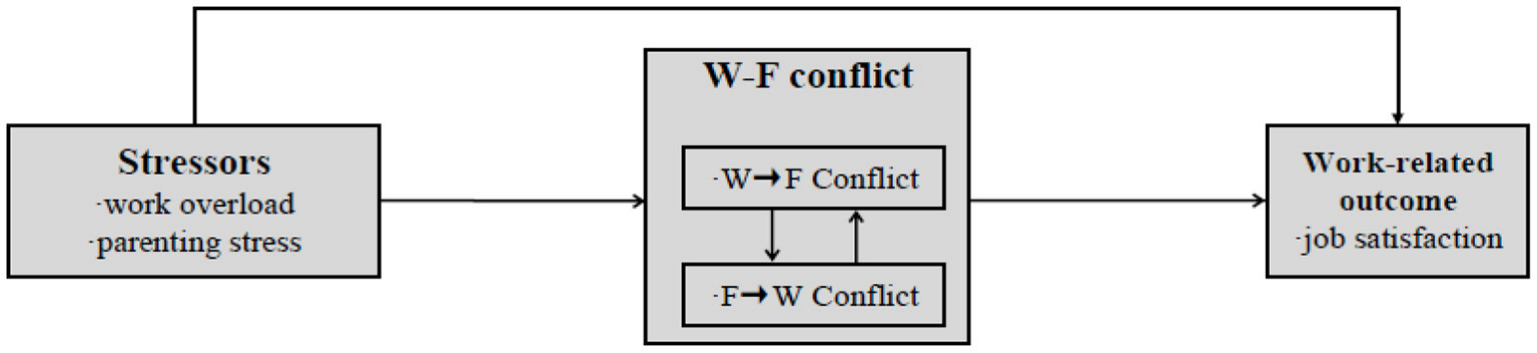
Model of stressors, work-family conflict and outcome.

Considering that there are two relations (i.e., cross-domain relations and matching-domain relations) between work-family conflict and job satisfaction, this study, based on the pre-school teachers under the COVID-19, combines the two viewpoints mentioned above. More specifically, work-to-family conflict caused by work overload is associated with job satisfaction, and family-to-work conflict caused by parenting stress is also related to job satisfaction. As mentioned above, work-to-family conflict and family-to-work conflict are reciprocal, so we assume that the two variables are related to each other in our model. See Figure 1 for an adaptation of the model described by Frone et al. (1992) and Amstad et al. (2011) and their colleagues, integrating the cross-domain relations and matching-domain relations.

To summarize, during the COVID-19 epidemic sweeping the world, this study aimed to examine pre-school teachers' work and parenting experiences, and the associations among work overload, parenting stress, work-family conflict, and job satisfaction in a sample of Chinese female pre-school teachers with young children. Specifically, the following research questions will guide this study: (a) What are pre-school teachers' work and parenting processes like during the COVID-19? (b) What are the relationships among pre-school teachers' work overload, parenting stress, work-family conflict, and job satisfaction?

## Methods

### Participants

Participants in the current study were randomly recruited from the National Training Program for Pre-school Teachers during the COVID-19 epidemic in May 2020 in China. In the current study, the participants were 718 female pre-school teachers with children. Most pre-school teachers are 30–49 years old (80.7%), and all of them had children, 73.2% of whom had one child. Among them, the majority of pre-school teachers are from public pre-schools (73.6%), and 70.6% come from urban areas. Teachers' years of experience were selected into shorter than 3 years (5.0%), 3–5 years (10.7%), 6–10 years (31.7%), 11–15 years (19.2%), and 16 years or above (33.2%). The educational accomplishment of pre-school teachers was as follows: 9.1% finished senior high school, 27.0% finished up-to-3-year college, and 63.9% finished 4-or more-year university or higher.

### Measures

#### Teacher Experience Questionnaire

This questionnaire was designed for the current study to investigate pre-school teachers' experience during the COVID-19. The items in the survey were rationally derived and covered the following two domains: (1) home-based online work involvement. Teachers reported whether they participated in home-based online work, and work content, and work feelings. (2) Parenting involvement. This mainly focused on the parenting labor division and parenting feelings.

#### Work Overload

Work overload was assessed using the Role Overload (Peterson et al., 1995). The Role Overload has been revised and used in China with good reliability and validity (Li and Zhang, 2009). The Role Overload consists of 5 items (e.g., “I feel overburdened in my role”). Teachers responded to the items on a 5-point Likert scale ranging from (1) strongly disagree to (5) strongly agree. In the current sample, Cronbach's α of the scale was 0.92. Confirmatory factor analysis showed that χ^2^/*df* = 1.92, RMSEA = 0.06, CFI = 0.95, TLI = 0.96.

#### Parenting Stress

Parenting stress was assessed using the 15-item Chinese Parenting Stress Index-Short Form (PSI-SF-15, Luo et al., 2019). The PSI-SF-15 consists of 15 items and 3 dimensions: parental distress (e.g., “I often have the feeling that I cannot handle things very well,” 5 items), difficult child (e.g., “My child seems to cry or fuss more often than most children,” 5 items), and parent-child dysfunctional interaction (e.g., “Sometimes I feel my child doesn't like me and doesn't want to be close to me,” 5 items). Teachers responded to the items on a 5-point Likert scale ranging from (1) strongly disagree to (5) strongly agree. In our study, Cronbach's α of the PSI-SF-15 and subscales for parental distress, difficult child, and parent-child dysfunctional interaction were 0.92, 0.92, 0.93, 0.93, respectively. Confirmatory factor analysis revealed that χ^2^/*df* = 1.87, RMSEA = 0.06, CFI = 0.96, TLI = 0.96.

#### Work-Family Conflict

Work-family conflict was assessed using the Negative Spillover Work to Family and Negative Spillover Family to Work from the Work-Family Spillover Scale (Grzywacz and Marks, 2000). The Work-Family Spillover Scale has been previously revised and used in China with good reliability and validity (Zeng and Yan, 2013). The Negative Spillover Work to Family and Negative Spillover Family to Work were used to measure work-family conflict and family-work conflict, which consists of 4 items, respectively. Response options ranged from 1 (strongly disagree) to 5 (strongly agree). A sample item for the Negative Spillover Work to Family is “Stress at work makes you irritable at home.” In the current study sample, this scale had a Cronbach's α of 0.86. A sample item for Negative Spillover Family to Work is “Stress at home makes you irritable at work.” In the current sample, this scale had a Cronbach's alpha of 0.72. Confirmatory factor analysis revealed that χ^2^/*df* = 1.92, RMSEA = 0.06, CFI = 0.95, TLI = 0.97.

#### Job Satisfaction

Job satisfaction was assessed using the General Employee Satisfaction Questionnaire (Tsui et al., 1992). This scale consisting of 6 items, such as “Are you satisfied with your relationships with your colleagues,” has demonstrated good psychometric properties in China (Ma et al., 2015). Response options ranged from 1 (strongly unsatisfied) to 5 (strongly satisfied), and this scale had a Cronbach's α of 0.72 in the current study. Confirmatory factor analysis revealed that χ^2^/*df* = 1.62, RMSEA = 0.05, CFI = 0.94, TLI = 0.96.

#### Covariates

Pre-school teachers were asked to report information on the pre-school type (0 = public pre-school, 1 = private pre-school), teachers' years of experience, wage, educational level (1 = senior high school, 2 = college, 3 = university or higher), pre-school education teacher qualification (0 = without, 1 = with), the number of children (0 = one child, 1 = more than one child) geographic region (1 = western region, 2 = central region, 3 = eastern region) and living region (0 = rural area, 1 = urban area). Previous studies have shown that these variables are related to job satisfaction (Evans, 2001; Klassen and Chiu, 2010; Jia et al., 2017). Thus, these variables were included as covariates in the current study.

### Procedures

To be eligible for survey participation, pre-school teachers had to be at least 20 years old (female below age 20 is considered the marriage of a minor in China), currently living in China with at least one child at most 12 years old. We focused on young children under 12 years old because infant- and child-care facilities, pre-schools, and primary schools were ordered to close during the COVID-19, significantly affecting parents (i.e., teachers) with children from this age group. Pre-school teachers provided consent to participate in the study after receiving information about the research objectives and being assured that the information collected would be used solely for research purposes. Using the Wenjuanxing, a professional online questionnaire collection platform in China, researchers send e-questionnaires to pre-school teachers, which contain the instructions and a packet of surveys including measures of demographics, work overload, parenting stress, work-to-family conflict, family-to-work conflict, and job satisfaction. The effective period of the electronic questionnaire was set as 2 weeks. Pre-school teachers can work on the questionnaires via smartphones or computers at any time in the 2 weeks. If pre-school teachers are interrupted during the course of the survey, they can continue to finalize the questionnaire at their convenience.

Two weeks later, 1,566 e-questionnaires were completed and returned. From the 1,566 completed questionnaires, we identified 718 female pre-school teachers, who have children. All the analyses in the current study were conducted based on data from the remaining 718 participants. All procedures performed in this study were in accordance with the ethical standards of and approved by the institutional review committee at the study's home institution. The research was carried out in accord with the ethical standards in the treatment of human participants and the work was approved by the Institutional Review Board at the study's home institution.

### Data Analysis

Descriptive and correlation statistics were analyzed in IBM SPSS Statistics version 22. Hypotheses were tested by conducting path analyses via Mplus Version 7.4. The current study built a path analyses model, which included two independent variables (i.e., work overload and parenting stress), two mediators (i.e., work-to-family conflict and family-to-work conflict), and one dependent variable (i.e., job satisfaction). Covariates were included in the model as exogenous variables. The following indexes recommended by Hu and Bentler (1999) and Kline (2011) were used to evaluate the fitness of the examined model to the data: a comparative fit index (CFI) and Tucker-Lewis index (TLI) above 0.95, a standardized root mean square residual (SRMR) below 0.10, and a root mean square error of approximation (RMSEA) below 0.08. The indirect effect model was tested using bootstrap (with 5,000 replicates) to calculate the 95% confidence interval (CI) and the indirect effect is significant when the CI does not include zero.

## Results

### Experience of Pre-school Teacher Home-Based Online Work During the COVID-19

**Figure 2:**
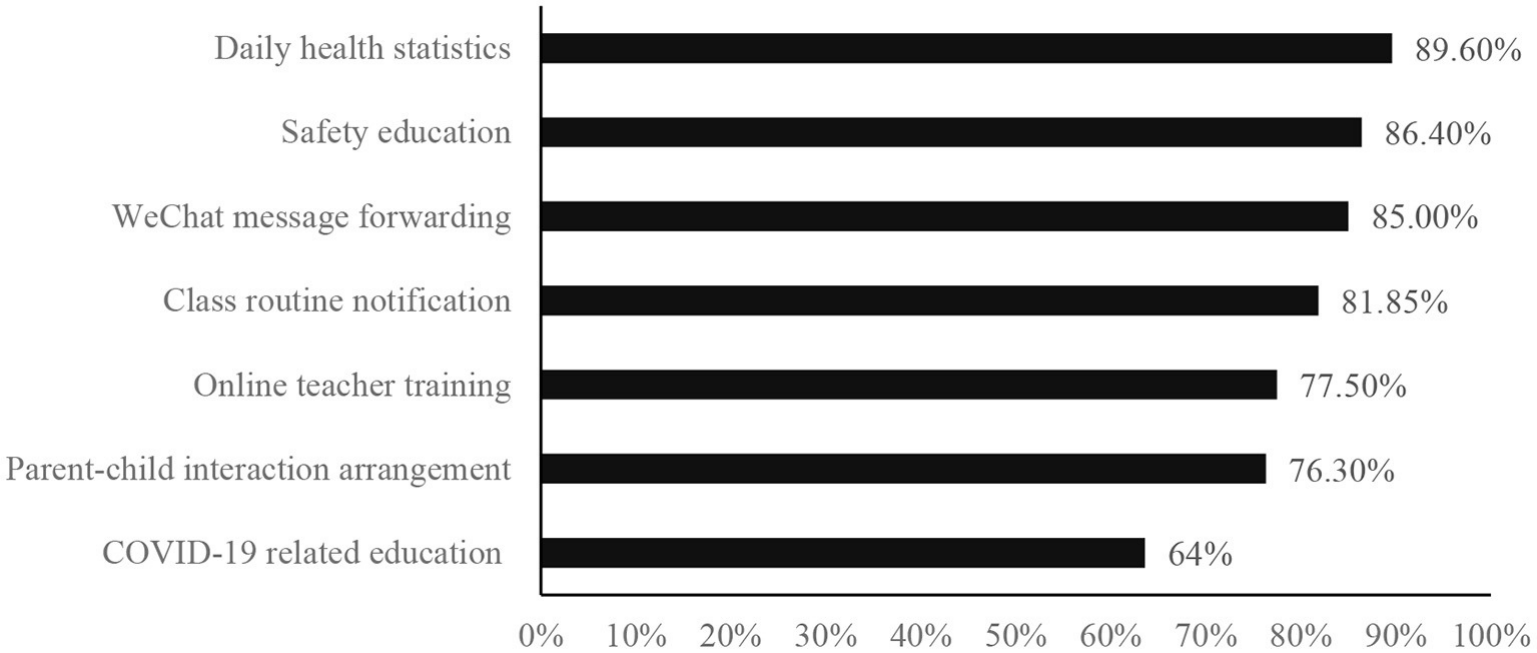
Pre-school teachers' work context during the COVID-19 epidemic.

Results indicated that pre-school teachers' working style, workload, and work content all changed during the COVID-19. In the current study, all pre-school teachers experienced home-based online work. Compared with offline work before the COVID-19 outbreak, 74.5% of pre-school teachers reported an increase in workload. And 39.6% of teachers reported that their workload has increased by more than 40.0%. As for the work content, pre-school teachers' work content was complex and varied (see Figure 2 for details). Among them, the daily health statistics and COVID-19 related education are new work content, which pre-school teachers have never experienced before. Although safety education, WeChat message forwarding, class routine notification, teacher training, and parent-child interaction arrangement are familiar to pre-school teachers, the specific working contents have changed significantly in the context of the epidemic. For example, class routine notifications include new information, that is, health protection during the COVID-19.

As for the parenting process, all pre-school teachers reported more child-care tasks than before the COVID-19 outbreak. Among them, 50.9% of pre-school teachers are the main caregiver and undertake the parenting task by themselves. As a mother, the main parenting tasks of a pre-school teacher were to take care of her children in daily life, to help them with their studies, and to spend leisure time with them. In addition, all the teachers believed that they were spending more time with their children during the COVID-19 outbreak than before. And, all of them agreed that parenting was more stressful than it was before the COVID-19 outbreak.

### Associations Among Work Overload, Parenting Stress, Work-Family Conflict, and Job Satisfaction

Table 1 contains descriptive statistics for the current study. As shown in Table 1, correlations among key study variables were statistically significant and in the expected directions. Work overload was negatively associated with job satisfaction and positively associated with work-to-family conflict and family-to-work conflict, respectively. The statistically significant correlations in the same direction were found among parenting stress and job satisfaction, work-family conflict, and family-work conflict. Also, work-to-family conflict was positively associated with family-to-work conflict. Correlation values range from 0.299 to 0.608. Using 0.1, 0.3, and 0.5 to represent “small,” “medium,” and “large” correlations (Cohen, 1988), the effect sizes of the correlation in the current study ranged from “medium” to “large” (see Table 1 for details).

**Table 1:** Correlations and descriptive statistics for the main study variables.

**Variables**	**1**	**2**	**3**	**4**	**5**
1. Work overload	–				
2. Parenting stress	0.403^***^	–			
3. W-F conflict	0.608^***^	0.476^***^	–		
4. F-W conflict	0.521^***^	0.578^***^	0.548^***^	–	
5. Job satisfaction	−0.348^***^	−0.253^***^	−0.354^***^	−0.299^***^	–
Skew	−0.161	0.044	0.024	0.224	−0.359
Kurtosis	−0.160	−0.412	−0.496	−0.570	0.250
Mean	3.153	2.854	2.828	2.564	3.213
Standard deviation	0.897	0.710	1.002	0.952	0.792

*W-F conflict, work-to-family conflict; F-W conflict, family-to-work conflict; children, number of children.*

*** *p < 0.001 (two-tailed)*.

**Figure 3:**
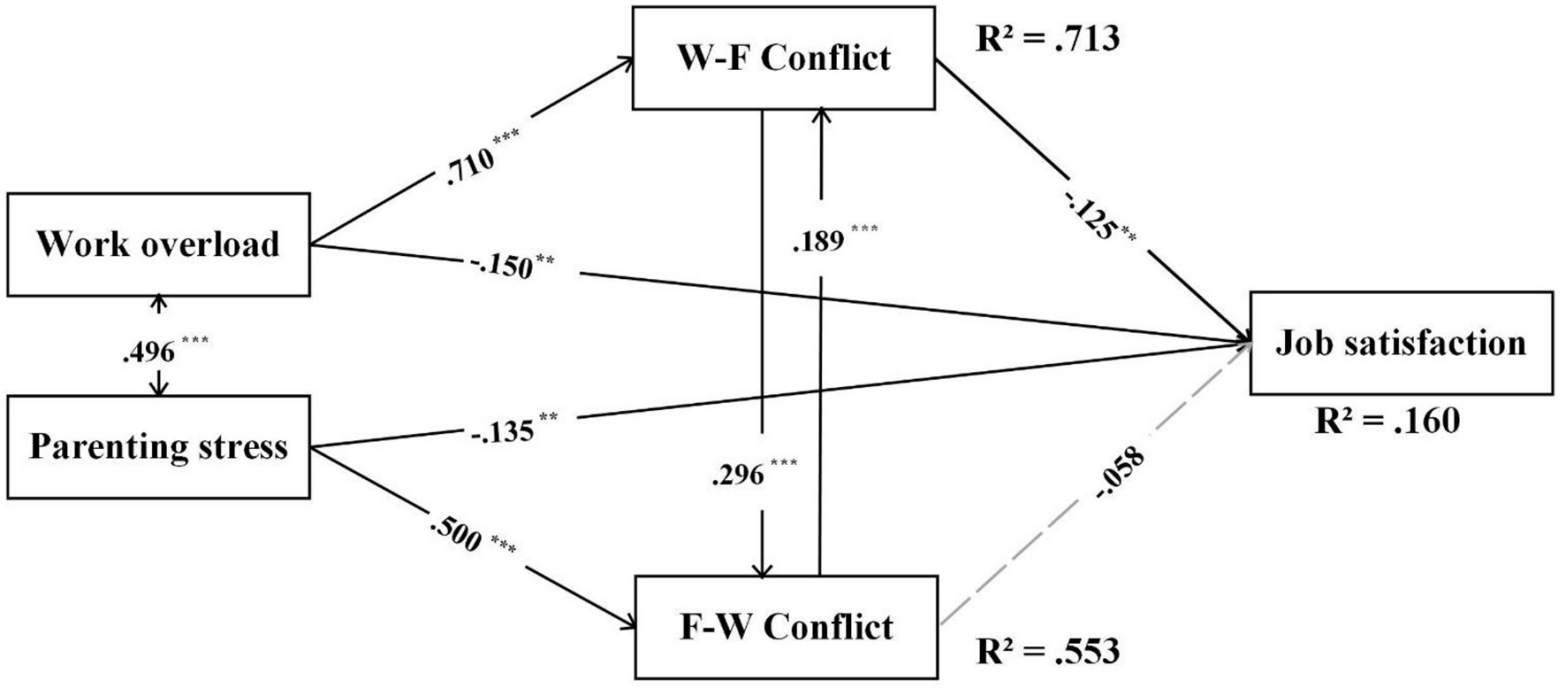
Indirect effect model among work overload, parenting stress, work-to-family conflict, family-to-work conflict, and job satisfaction. W-F conflict, work-to-family conflict; F-W conflict, family-to-work conflict. All the reported parameters are standardized. For clarity, covariates are not shown in the figure. **p < 0.01, ***p < 0.001 (two-tailed).

Figure 3 showed the indirect effect analysis among work overload, parenting stress, work-to-family conflict, family-to-work conflict, and job satisfaction. The models fit the data adequately [χ^2^/*df* = 1.747; CFI = 0.996, TLI = 0.980, SRMR = 0.045, RMSEA = 0.035 with 90% CI (0.000, 0.059)]. As shown in Figure 2, work overload and parenting stress were both negatively associated with job satisfaction (β = −0.150, *p* < 0.01, β = −0.135, *p* < 0.01, respectively), and positively associated with one aspect of work-family conflict, respectively. Specially, work overload was positively associated with work-to-family conflict (β = 0.710, *p* < 0.001), and parenting stress was positively associated with family-to-work conflict (β = 0.500, *p* < 0.001). The work-to-family conflict was negatively associated with job satisfaction (β = −0.125, *p* < 0.001), but family-to-work conflict was not significantly associated with it (β = −0.058, *p* > 0.05). Regarding the two aspects of work-family conflict, the positively bidirectional associations were found (β = 0.296, *p* < 0.001, β = 0.189, *p* < 0.001, respectively).

Bootstrapping analyses identified 2 indirect pathways linking work overload, parenting stress, work-to-family conflict, family-to-work conflict, and job satisfaction (see Table 2): (1) work overload was negatively associated with job satisfaction via its positive association with work-to-family conflict [95% CI (0.019, 0.029), standardized estimate = 0.089]. (2) Parenting stress was negatively associated with job satisfaction via the serial mediation of family-to-work conflict and work-to-family conflict [95% CI (0.140, 0.002), standardized estimate = 0.012].

**Table 2:** The bootstrap confidence interval and effect size of the mediation model.

**Path**	**Estimate**	**95% CI**
Work overload → W-F conflict → job satisfaction	0.089	(0.019, 0.029)
Parenting stress → F-W conflict → job satisfaction	0.029	(−0.010, 0.017)
Parenting stress → W-F conflict → F-W conflict → job satisfaction	0.012	(0.140, 0.002)

*W-F conflict, work-to-family conflict; F-W conflict, family-to-work conflict*.

## Discussion

As part of the isolation measure, the Chinese government has closed all types of schools, including pre-school s for nearly half a year, from January 2020 to May 2020. The goal of the isolation measure is to reduce the transmission of COVID-19 in the community. However, for female pre-school teachers with children, this period has been a challenging experience. They need to educate others' young children remotely and take care of their own children at home. This study focuses on female pre-school teachers who work and raise their children at home during the epidemic. Pre-school teachers in the current study reported that COVID-19 has increased work overload and parenting stress. Two significant indirect associations were found among work overload, parenting stress, work-family conflict, and job satisfaction. More specifically, work overload was negatively associated with job satisfaction via its positive association with work-to-family conflict. And parenting stress was negatively associated with job satisfaction via both family-to-work conflict and work-to-family conflict. This section will discuss these findings and their implications for future studies and practical improvements.

### Challenging Experience During Home-Based Online Work

As the workplace was transferred to the home, the way pre-school teachers work has also changed to online work. In the past, online education was only an auxiliary way of face-to-face education, which was not carried out on a large scale. During the COVID-19, however, pre-school teachers do most of their work online, which is unfamiliar to pre-school teachers. Studies in other cultures have also found that teachers face difficulties with the use of online education technologies (Knig et al., 2020), which supports our study's findings to some extent. In addition to the working platform, the unfamiliarity and increased work content are also challenging for the teaching profession. As this study showed, pre-school teachers had many new and special tasks in the epidemic, such as daily health statistics and COVID-19 related education. Although these are not the routine work content before the COVID-19 outbreak, pre-school teachers have to do these to check the health status of their students during the COVID-19.

As for pre-school teachers also reporting an increase in parenting stress, here are two possible explanations. One possible reason is that long hours at home allow pre-school teachers to devote more time with their children, which increases the parenting tasks. Under the COVID-19 epidemic, pre-school teachers' young children are also forced to stay at home and they need parents' daily care and company. As such, pre-school teachers are likely to spend more hours a day with children, either actively or passively, than before. Long-term parenting can leave pre-school teachers with low energy and high burnout. Another possible reason is that the COVID 19 epidemic may increase anxiety and depression in children and mothers, which may increase parenting challenges. Both children and adults are psychologically affected by the COVID-19 pandemic. For children, they experience fears, uncertainties, substantial changes to their routines alongside high physical and social isolation (Imran et al., 2020). As adults, pre-school teachers, especially female pre-school teachers, may also experience psychological challenges. A Chinese study of 88,611 teachers found that the prevalence of anxiety was higher for female teachers than male teachers (Li et al., 2020). When anxious female pre-school teachers take on the role of mother, caring for children experiencing fear, their parenting stress may be even greater.

The results of pre-school teachers' experience provide us with a vivid picture of the living status of the teaching profession under the epidemic situation. Overall, the results showed that pre-school teachers' work overload and parenting stress were higher than they were before the COVID-19 outbreak.

### Associations Among Work Overload, Parenting Stress, Work-Family Conflict, and Job Satisfaction

The findings of this study confirm Hypothesis 1. Namely, during the COVID-19 epidemic, pre-school teachers' work-to-family conflict and family-to-work conflict are associated with each other. This proposed hypothesis is consistent with Frone et al. (1997) viewpoint. Our finding also supported the results of existing empirical studies (Huang et al., 2004). In other words, during the COVID-19, if the pre-school teacher's work interfered family, the conflict would in turn affect the resources in the family domain, resulting in family-to-work conflict. On the contrary, this path also works.

As assumed, we found that work overload was negatively associated with job satisfaction. The findings supported previous studies' results that there is a negative relationship between work overload and job satisfaction (Cheng and Ren, 2010; Altaf and Wan, 2011). It is worth noting that this study differs from previous studies. Especially, the samples of this current study are female pre-school teachers during the COVID-19 epidemic, who are participants from a particular social context. Influenced by the epidemic, distance teaching has become the mainstream of the education form, which forced pre-school teachers to face unprecedented online teaching stress. Work overload negatively influenced pre-school teachers' work attitudes, thus resulting in a decrease in job satisfaction (Klassen and Chiu, 2010; Sheraz et al., 2014).

Meanwhile, the results also indicated that parenting stress had a direct negative association with job satisfaction. Although existing studies have focused more on the impact of work-related stress on job satisfaction, others have suggested that parenting stress may have a potentially negative impact on job satisfaction. Kline et al. (1991) hold that family-related duties and stress spill over from one family domain to the work domain, which supports the current study results. Teacher's parenting place and workplace overlap during the home-based work (Riegler et al., 2020; Spinelli et al., 2020). As such, teachers' parenting stress is more likely to immerse to work domain, weakening teachers' active work emotion. In other words, a mother, struggling with parenting struggles, could find it difficult to actively interact with children and colleagues online. It could eventually lead to pre-school teachers being dissatisfied with their job.

Also, results found that work overload was negatively associated with job satisfaction which was positively associated with work-to-family conflict. This finding is consistent with prior extensive research before the COVID-19 outbreak that showed that work-family conflict plays a mediating role between job stress and job satisfaction (Ford et al., 2007). Specifically, unprecedented work overload under the COVID-19, such as online teaching maladjustment and technical difficulties, could make pre-school teachers incompetent for work roles and take up resources in other areas, such as parenting. During the COVID-19, work and family are at a weak boundary, with high permeability and flexibility between the two fields (Clark, 2000). Therefore, work stress is easy to infiltrate into the family, resulting in work-to-family conflict. Influenced by Confucian culture, the family is the cornerstone of Chinese society, and the Chinese value family interests more (Michailova and Hutchings, 2006). Chinese pre-school teachers would give priority to their families and work to realize their family's interests. Adhering to family-centered values, pre-school teachers may attribute work-to-family conflict to work rather than family when work interferes with parenting. Finally, pre-school teachers may express dissatisfaction with their work.

Results showed that parenting stress was negatively associated with job satisfaction via a positive association with family-to-work conflict and work -to- family conflict. That is, family-to-work conflict and work-to-family conflict co-play a serial mediation between parenting stress and job satisfaction. Under the epidemic situation, pre-school teachers are incompetent for parenting roles and experience more parenting stress (Reitman et al., 2002), due to having limited time and energy. Based on border theory (Ashforth et al., 2000), parenting stress leads to pre-school teachers' tendency to seize the resources originally allocated to work, thus the parenting interference with work. Interdomain transitions between work and family are conceptualized as bidirectional: individuals can transit from work to family and from family to work (Matthews et al., 2013). According to this notion, family-to-work conflict would spill over further into work-to-family conflict. In other words, if pre-school teachers are unable to perform their work tasks as a result of parenting, these unfulfilled work tasks would in turn hinder the smooth development of parenting. In this case, pre-school teachers' job satisfaction decreases after work intrudes on their families.

It should be noted that work-to-family conflict was associated with job satisfaction, while the relationships between family-to-work conflict and job satisfaction were not significant. This supports the matching hypothesis rather than the cross-domain hypothesis between work-family conflict and work-domain outcome variables (Amstad et al., 2011). That is, the work-family conflict could show stronger relationships to same-domain outcomes than to cross-domain outcomes. This result has a unique explanation in the context of Chinese culture. Chinese teachers may still be satisfied with their jobs when families interfere with their job. Chinese cultures are “collective and familial” (Hofstede and Bond, 1988). As such, Chinese teachers believe that family interests are higher than work, and regard work as a way to support family development. Therefore, Chinese teachers may not be dissatisfied with their jobs when families interfere with them. At the same time, influenced by the concept of gender division of labor in China, women are expected to assume the responsibilities for the family and children. Female pre-school teachers, as educators and mothers, are more likely to regard the family as their primary responsibility in life, and teachers are less likely to complain and be dissatisfied with their work when family interferes work.

### Practical Implications and Limitations

The current findings have practical implications in understanding and improving pre-school teachers' online teaching experience at home during the social quarantine period. Although the epidemic in China has been greatly controlled, the regular prevention efforts have not slowed down and people may still be infected in some areas. It is necessary to make clear specific measures to relieve pre-school teachers' work stress and improve their job satisfaction during the COVID-19. First, efforts to reduce pre-school teachers' work demands could be made. During the COVID-19, flexible work arrangements, including flexible time schedules and locations, compressed workweeks, and flexible shift work, could be implemented in pre-schools (Thompson et al., 2015; Moreira et al., 2019). Pre-school education managers should reasonably set all kinds of missions such as health information statistics, online resource collection, and so on, to avoid duplication of the task and bring extra work demands to teachers. Second, pre-school teacher professional development programs initiated by the government and pre-schools are also effective strategies to improve pre-school teachers' work-family balance and job satisfaction. A promising example of such a method is the Connected Educators Project for educators by the U. S. Department of Education Office of Educational Technology (2013). Considering that the epidemic is not over and online teaching at home may continue, it is considered to provide training programs in online teaching for pre-school teachers.

The current study has certain limitations. First, only the questionnaires were employed because we are subject to “keeping a social distance.” Future methods are needed to get more objective data, such as interviews and observations. Second, our study did not consider the larger sample size and more diverse teacher characteristics (i.e., race/ethnicity, gender, area). Due to China's vast territory and the imbalance of pre-school teachers' living context, it would be helpful to take into account diverse samples with various background information. Third, future research can also add other external variables, such as social support and stress coping styles, into the model to gain a more comprehensive understanding of the factors influencing pre-school teachers' career and parenting experience (Miloseva et al., 2017). Fourth, although we controlled for teacher-related and region-related covariates, we did not focus on school-related variables. Organization-related variables, such as principals' leadership and organizational climate, are associated with job satisfaction (Cerit, 2009; Ghavifekr and Pillai, 2016). Future studies could fully consider covariates. Finally, changes in pre-school teachers' work experience are only based on their subjective recall. We did not collect the data about pre-school teachers' work overload and parenting stress before the COVID-19 outbreak.

## Conclusions

This study contributes to understanding pre-school teachers' work overload and parenting stress during the COVID-19 and how work-family conflict caused by work overload and parenting stress are related to job satisfaction. In the current study, Chinese pre-school teachers reported that the COVID-19 has increased work overload and parenting stress. Work overload and parenting stress were negatively associated with job satisfaction via its positive association with work-family conflict. Given the importance of work-family balance for pre-school teachers' job satisfaction, identifying practical programs and prescriptions by which to relieve stressors from work and family is essential. Based on Chinese pre-school teachers, this study provides preliminary insights and enlightenment for improving the personal and professional development environment of pre-school teachers under the epidemic.

## Data Availability Statement

The raw data supporting the conclusions of this article will be made available by the authors, without undue reservation.

## Ethics Statement

The studies involving human participants were reviewed and approved by Institute of Early Childhood Education, Faculty of Education, Beijing Normal University. The patients/participants provided their written informed consent to participate in this study. Written informed consent was obtained from the individual(s) for the publication of any potentially identifiable images or data included in this article.

## Author Contributions

XH and QL contributed equally to the preparation of this article, designed the research, and wrote the manuscript. MZ was mainly responsible for the introduction and implications. All authors contributed to the article and approved the submitted version.

## Conflict of Interest

The authors declare that the research was conducted in the absence of any commercial or financial relationships that could be construed as a potential conflict of interest.
